# Comparative 'omics analyses differentiate *Mycobacterium tuberculosis* and *Mycobacterium bovis* and reveal distinct macrophage responses to infection with the human and bovine tubercle bacilli

**DOI:** 10.1099/mgen.0.000163

**Published:** 2018-03-20

**Authors:** Kerri M. Malone, Kévin Rue-Albrecht, David A. Magee, Kevin Conlon, Olga T. Schubert, Nicolas C. Nalpas, John A. Browne, Alicia Smyth, Eamonn Gormley, Ruedi Aebersold, David E. MacHugh, Stephen V. Gordon

**Affiliations:** ^1^​ UCD School of Veterinary Medicine, University College Dublin, Belfield, Dublin 4, Ireland; ^2^​ Animal Genomics Laboratory, UCD School of Agriculture and Food Science, University College Dublin, Belfield, Dublin 4, Ireland; ^3^​ Department of Biology, Institute of Molecular Systems Biology, ETH Zurich, Zurich CH-8093, Switzerland; ^4^​ Tuberculosis Diagnostics and Immunology Research Centre, UCD School of Veterinary Medicine, University College Dublin, Belfield, Dublin 4, Ireland; ^5^​ UCD Conway Institute of Biomolecular and Biomedical Research, University College Dublin, Belfield, Dublin 4, Ireland; ^6^​ UCD School of Medicine, University College Dublin, Dublin 4, Ireland; ^7^​ UCD School of Biomolecular and Biomedical Science, University College Dublin, Dublin 4, Ireland; ^†^​ Present address: European Bioinformatics Institute (EMBL-EBI), Wellcome Genome Campus, Hinxton, Cambridge CB10 1SD, UK.; ^‡^​ Present address: Kennedy Institute of Rheumatology, Nuffield Department of Orthopaedics, Rheumatology and Musculoskeletal Sciences, University of Oxford, Headington, Oxford OX3 7FY, UK.; ^§^​ Present address: Department of Human Genetics, University of California, Los Angeles, USA.; ^¶^​ Present address: Quantitative Proteomics and Proteome Centre Tübingen, Interfaculty Institute for Cell Biology, University of Tübingen, 72076 Tübingen, Germany.

**Keywords:** tuberculosis, *Mycobacterium bovis*, macrophage, transcriptomics, proteomics

## Abstract

Members of the *Mycobacterium tuberculosis* complex (MTBC) are the causative agents of tuberculosis in a range of mammals, including humans. A key feature of MTBC pathogens is their high degree of genetic identity yet distinct host tropism. Notably, while *Mycobacterium bovis* is highly virulent and pathogenic for cattle, the human pathogen *M. tuberculosis* is attenuated in cattle. Previous research also suggests that host preference amongst MTBC members has a basis in host innate immune responses. To explore MTBC host tropism, we present in-depth profiling of the MTBC reference strains *M. bovis* AF2122/97 and *M. tuberculosis* H37Rv at both the global transcriptional and the translational level via RNA-sequencing and SWATH MS. Furthermore, a bovine alveolar macrophage infection time course model was used to investigate the shared and divergent host transcriptomic response to infection with *M. tuberculosis* H37Rv or *M. bovis* AF2122/97. Significant differential expression of virulence-associated pathways between the two bacilli was revealed, including the ESX-1 secretion system. A divergent transcriptional response was observed between *M. tuberculosis* H37Rv and *M. bovis* AF2122/97 infection of bovine alveolar macrophages, in particular cytosolic DNA-sensing pathways at 48 h post-infection, and highlights a distinct engagement of *M. bovis* with the bovine innate immune system. The work presented here therefore provides a basis for the identification of host innate immune mechanisms subverted by virulent host-adapted mycobacteria to promote their survival during the early stages of infection.

## Data Summary

1. RNA-seq datasets have been deposited in ENA; accession number PRJEB23469.

2. SWATH MS data and OpenSWATH outputs have been deposited in PeptideAtlas under identifier PASS00685 (http://www.peptideatlas.org/PASS/PASS00685).

**Impact Statement**

The *Mycobacterium tuberculosis* complex (MTBC) includes the most important global pathogens for humans and animals, namely *Mycobacterium tuberculosis* and *Mycobacterium bovis*, respectively. These two exemplar mycobacterial pathogens share a high degree of genetic identity, but the molecular basis for their distinct host preference is unknown. In this work we integrated transcriptomic and proteomic analyses of two reference strains, *M. tuberculosis* H37Rv and *M. bovis* AF2122/97, to elucidate global quantitative differences between them at the mRNA and protein level. We then integrated these data with transcriptome analysis of the bovine macrophage response to infection with either pathogen. Increased expression of the ESX-1 virulence system in *M. bovis* AF2122/97 appeared a key driver of an increased cytosolic nucleic acid sensing and IFN response in bovine macrophages infected with *M. bovis* AF2122/97 compared to *M. tuberculosis* H37Rv. Our work demonstrates the specificity of host–pathogen interaction and how the subtle interplay between mycobacterial phenotype and host response may underpin host specificity amongst MTBC members.

## Introduction

The *Mycobacterium tuberculosis* complex (MTBC) comprises ten mycobacterial species that cause tuberculosis (TB) in a broad range of mammalian species, including humans [1–4]. Typically, MTBC species show greater than 99 % nucleotide sequence similarity and yet exhibit distinct host preference, indicating that this low level of genetic divergence holds major implications for host–pathogen interactions [1–3]. Divergence in host tropism is illustrated through the comparison of the human-adapted *Mycobacterium tuberculosis* with the animal bacillus *Mycobacterium bovis. M. tuberculosis* is a highly successful pathogen and is the world’s leading cause of death from an infectious agent with 1.7 million deaths reported in 2016 [5]. *M. bovis* predominantly causes disease in cattle and bovine TB exacts a tremendous economic burden through production loss and control costs [6–8]. *M. tuberculosis* appears unable to sustain (i.e. through cycles of infection, disease and transmission) in non-human animal populations, a fact that has been confirmed using an experimental bovine infection model [1, 9]: while cattle infected with *M. bovis* display characteristic pathology, cattle infected with *M. tuberculosis* show minimal pathology despite positive skin-test and IFN-γ responses indicative of successful infection. Conversely, while *M. bovis* can both infect humans and cause pulmonary disease that is clinically indistinguishable from *M. tuberculosis*, it rarely transmits among immunocompetent hosts [10, 11].

On a cellular level, the alveolar macrophage is the frontline host immune cell that encounters both *M. tuberculosis* and *M. bovis*, and its role during early-stage infection is well established [12–18]. Several studies have highlighted significant differences in the production of key innate factors, chemokines and cytokines at both the transcript and the protein level in macrophages infected with *M. tuberculosis* or *M. bovis* [15–20]. However, these studies evaluated only a subset of the innate response in macrophages, and differences in the global transcript and protein response to infection with *M. tuberculosis* or *M. bovis* remains unknown. The central role of the alveolar macrophage during infection is also reflected in the fact that pathogenic mycobacteria have evolved several immune-evasion strategies to circumvent the killing mechanisms of the macrophage, including inhibition of phagosomal maturation, phagosomal escape and suppression of innate immune signalling [12–18]. This facilitates the dissemination of the bacilli to other macrophages and ultimately throughout the host, with the concomitant development of immunopathology. Transmission of infection then occurs through the rupture of lesions into associated airways and the dispersal of bacilli [17, 18]. Thus, it can be hypothesized that the initial interaction between host and pathogen may be key for the host preference observed between *M. tuberculosis* and *M. bovis*; whether this interaction has roots in host-centric or pathogen-centric processes, or indeed a combination of both, has yet to be fully elucidated.

*M. tuberculosis* H37Rv and *M. bovis* AF2122/97 were the first MTBC genomes to be fully sequenced and they represent the default reference strains for the human and animal tubercle bacilli [2, 21, 22]. It was hypothesized that host tropism between these two species may be explained by differential gene expression profiles as a result of low genetic divergence [2, 21, 22]. So far, functional studies have revealed that genetic changes between the two pathogens are responsible for differential nitrate reductase activity, for the loss of phenolic glycolipid production in *M. tuberculosis* H37Rv in contrast to *M. bovis,* and for differences in the PhoPR regulation system that governs the expression of virulence-related pathways such as EsxA/ESAT-6 secretion and cell wall lipid biosynthesis [23–26]. While these studies highlight important differences between the two pathogens, host tropism likely involves a combination of events that affect the expression and regulation of multiple virulence-associated factors and/or the transcriptional regulators that govern their activity. In 2007, two microarray-based studies highlighted multiple genes that were differentially expressed between *M. bovis* and *M. tuberculosis*, including genes encoding the major antigens MPT83 and MPT70 that were expressed at higher levels in *M. bovis*, and genes involved in SL-1 production that were expressed at a higher level in *M. tuberculosis* [27, 28]. Since these reports, investigations into species-specific expression profiles of the two pathogens have been lacking at the global transcriptional and proteomic levels. Definition of the differential ‘expressome’ between *M. tuberculosis* and *M. bovis* will shed light on how alternate expression of two highly related genomes impacts on the ultimate success of these pathogens and provide insight into host specificity within the MTBC.

As a route to defining host preference between *M. bovis* and *M. tuberculosis*, we have conducted in-depth profiling of *M. bovis* AF2122/97 and *M. tuberculosis* H37Rv at both the global transcriptional and the translational level *in vitro* using RNA-sequencing (RNA-seq) and Sequential Window Acquisition of all Theoretical Spectra (SWATH) MS, a massively parallel targeting MS that provides highly reproducible quantitative measurements across samples [29]. To address how MTBC pathogen variation impacts on the host innate response, we have performed detailed comparative transcriptomic analyses of the bovine alveolar macrophage response to infection with both pathogens using RNA- seq. Through these analyses, we reveal significant differential expression of virulence-associated pathways between *M. tuberculosis* H37Rv and *M. bovis* AF2122/97, in particular the ESX-1 secretion system, while the macrophage infection study highlighted a distinct engagement of *M. bovis* AF2122/97 with the bovine innate immune system, in particular with the cytosolic DNA-sensing pathways of the macrophage.

## Methods

### Mycobacterial culture for pathogen transcriptomics and proteomics

Exponentially grown mycobacterial liquid cultures were established in Sauton’s basal media with 10 mM pyruvate and 0.025 % tyloxapol. For mid- to late-log phase culture, mycobacterial cells were grown to an optical density (OD_600_) of 0.6–0.8 at 37 °C prior to harvest. For the current study, six *M. bovis* AF2122/97 and six *M. tuberculosis* H37Rv replicates were prepared. Matched RNA and protein samples were harvested and prepared for strand-specific RNA-seq or SWATH MS.

### RNA extraction, RNA-seq library preparation and high-throughput sequencing for *M. bovis* AF2122/97 and *M. tuberculosis* H37Rv

Mycobacterial cells were harvested by centrifugation at 2500 ***g*** for 10 min and the pellet was re-suspended in 1 ml of TRIzol reagent (Life Technologies). The suspension was transferred to a 2 ml screw cap tube and the cells were lysed by bead-beating for 30 s at maximum setting using 1 mm glass beads (Sigma) on a MagNaWLyser instrument (Roche). Samples were placed at 80 °C immediately and thawed before use. Then, 20 % (v/v) chloroform was added, the sample were shaken vigorously for 15 s and incubated for 2–3 min at room temperature. The samples were centrifuged at 12 000 ***g*** for 15 min at 4 °C and the top phase was added to the DNA-free columns from the RNeasy plus kit (Qiagen). The samples were processed as per the manufacturer’s guidelines with the following exceptions: 1.5 volumes of 100 % ethanol was added to the sample prior to its application to the RNeasy column in order to recover all RNA species. RNA was eluted in molecular-grade water and its concentration was determined using a NanoDrop spectrophotometer (NDW1000) prior to DNase treatment. A DNase treatment using a TURBO DNase kit (Thermo Fisher Scientific) was performed by following the vigorous DNase treatment as per the manufacturer’s guidelines: 7 µl of DNase buffer + 1 µl enzyme at 37 °C for 30 min followed by a further 1 µl of enzyme and incubation at 37 °C for 30 min. A DNase stopping solution was not added to the samples as they were column-purified and concentrated using the RNA Clean and Concentrator kit according to the manufacturer’s guidelines (Zymo). RNA was eluted in molecular-grade water and its concentration was determined using a NanoDrop spectrophotometer (NDW1000). RNA integrity number (RIN) values were assessed for each RNA sample being considered for RNA-seq using a 2100 Bioanalyser and RNA 6000 Nano kit (both Agilent) according to the manufacturer’s guidelines. RIN values are calculated by assessing the entire electrophoretic trace of an RNA sample, along with the 23S/16S rRNA intensity value. Only samples with a RIN value >8 were selected for further analysis by RNA-seq. Sequencing libraries were prepared at the Genomics Core, Michigan State University, Michigan, USA, using the Illumina Truseq Stranded Total RNA Library Prep Kit LT and the Epicenter RiboWZero Magnetic Bacteria kit to deplete rRNA. Single-end, strand-specific 50 bp read data were produced with base calling performed by using Illumina Real Time Analysis (RTA) v1.18.64 (Illumina HiSeq 2500).

### Differential gene expression analysis of *M. bovis* AF2122/97 and *M. tuberculosis* H37Rv RNA-seq data

Computational analyses were performed on a 32-node Compute Server with Linux Ubuntu (version 12.04.2). Briefly, adapter sequence contamination and paired-end reads of poor quality were removed from the raw data. At each step, read quality was assessed with FastQC (version 0.10.1) [30]. Single-end reads were aligned to the *M. bovis* AF2122/97 or *M. tuberculosis* H37Rv reference genomes with the aligner Stampy in hybrid mode with the BWA algorithm [31]. Read counts for each gene were calculated using featureCounts, set to unambiguously assign uniquely aligned single-end reads in a stranded manner to gene exon annotation [32].

**Fig. 1.**
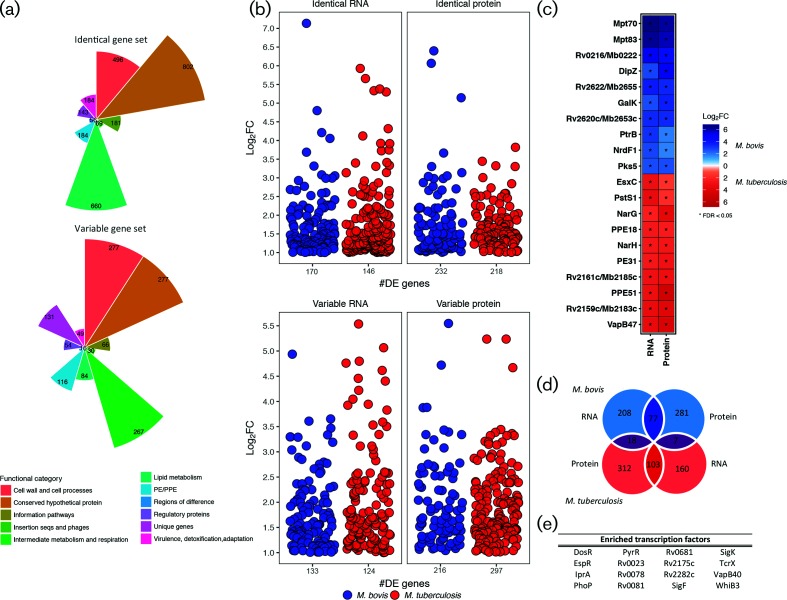
(a) The number of genes from *M. bovis* AF2122/97 and *M. tuberculosis* H37Rv classified as either ‘Identical’ (100 % conserved in length and amino acid sequence, top plot) or Variable (all other orthologous genes, bottom plot). Colours represent the various gene categories to which each gene belongs. (b) The level of expression (‘log_2_FC’) of Identical genes (top panel) and Variable genes (bottom panel) that are differentially expressed (|log_2_FC|>1, FDR<0.05) and upregulated in either *M. bovis* AF2122/97 (blue) or *M. tuberculosis* H37Rv (red) at both the RNA and the protein level. The number of genes in each category is indicated on the *x*-axis (‘#DE genes’). (c) The top 20 differentially expressed genes at the RNA and protein level [|log_2_FC|>1, FDR<0.05 (‘*’)] that are upregulated in *M. bovis* AF2122/97 (blue) or *M. tuberculosis* H37Rv (red). (d) The overlap of genes that are upregulated in either *M. bovis* AF2122/97 (blue) or *M. tuberculosis* H37Rv (red) at the RNA and protein level. Dark blue overlap represents those genes upregulated in *M. bovis* AF2122/97 only, dark red overlap represents those genes upregulated in *M. tuberculosis* H37Rv while purple overlaps represent those genes that show discordant expression patterns at the RNA and protein level between the two species. (e) The transcription factors enriched for ‘Identical’ genes (100 % conserved in length and amino acid sequence between the two species) that are differentially expressed between *M. bovis* AF2122/97 and *M. tuberculosis* H37Rv (Rand Index, *P*<0.01).

Prior to cross-species differential expression analysis, an Identical/Variable gene dataset was constructed for *M. tuberculosis* H37Rv and *M. bovis* AF2122/97 where orthologous genes were separated into those whose protein products are of equal length and 100 % conserved at the amino acid level (Identical, *n*=2775) from those that are not (Variable, *n*=1224) (Fig. 1a and Table S1, available in the online version of this article). Among the Variable genes are examples of truncated genes, genes that have been split into two or more as a result of in-frame sequence variance (leading to some genes being represented in more than one Variable gene pair), or genes that differ by a non-synonymous SNP resulting in an amino acid change at the protein level. Negative binomial modelling tools such as DESeq2, which was used in this instance, assume equal feature lengths when calculating differential expression (DE) of a gene, or in this case between orthologous genes of two species in a given condition [33]. For those annotations whose gene lengths are not equal, such as in the truncated/elongated/frameshift instances found in the *M. bovis* AF2122/97 genome with respect to *M. tuberculosis* H37Rv, analysis with DESeq2 would result in erroneous differential expression results; thus, a separate differential expression analysis was carried out for Variable genes using transcript per million (TPM) values that are normalized for feature length [33, 34]. Differential gene expression analysis for those genes of equal lengths was performed using the DESeq2 pipeline, correcting for multiple testing using the Benjamini–Hochberg method [33]. All further reference to DE genes between the two mycobacterial species will be with regard to a gene being expressed at a higher level in one species with respect to the other, and hence if a gene is upregulated in *M. bovis* AF2122/97 it is downregulated or expressed at a lower level in *M. tuberculosis* H37Rv and vice versa [log_2_ fold change |log_2_FC>1|, false discovery rate (FDR) threshold of significance <0.05].

### Transcription factor enrichment analysis

Data relating to the shift in the transcriptional landscape of *M. tuberculosis* upon overexpression of 183 transcription factors were used to perform a formal transcription factor enrichment analysis [35–37]. These data represent 9335 regulatory events and provide evidence for the control of over 70 % of the annotated genes in the *M. tuberculosis* H37Rv genome (FC>2, *P*<0.01) [35–37]. These data were analysed alongside the DE genes identified in this study between *M. bovis* AF2122/97 and *M. tuberculosis* H37Rv. Only genes and transcription factors that are 100 % identical in sequence and length between the two species were considered for this analysis. Over-representation of a transcription factor with a given set of DE genes was assessed by gene-regulon association and calculation of the Rand Index (log_2_FC>1 and *P*<0.05 for a given DE gene).

### Protein extraction and SWATH MS for *M. bovis* AF2122/97 and *M. tuberculosis* H37Rv

Mycobacterial cells were harvested by centrifugation at 2500 ***g*** for 10 min and the pellet was re-suspended in 1 ml LB buffer (0.1 M ammonium bicarbonate buffer, 8 M urea, 0.1 % *Rapi*GEST SFl;Waters). The suspension was transferred to a 2 ml screw cap tube and the cells were lysed by bead-beating for 30 s at maximum setting using 3 mm glass beads (Sigma) and a MagNaLyser instrument (Roche). The lysate supernatant was harvested by centrifugation at 12 000 ***g*** for 10 min and transferred to a clean 1 ml tube. The remaining pellet was re-suspended in LB buffer and the bead-beating cycle was repeated twice more. Protein lysate samples were stored at −80 °C. Protein samples were removed from −80 °C storage and thawed on ice. Total protein content was measured using the Qubit Protein Assay kit according to the manufacturer’s guidelines and protein concentrations were adjusted to 0.5 mg ml^−1^. Protein disulphide bonds were reduced by addition of 0.2 M Tris(2-carboxyethyl)phosphine (TCEP) and the resulting free cysteine residues were alkylated by addition of 0.4 M iodoacetamide (IAA). Extracted protein samples were diluted with 0.1 M ammonium bicarbonate buffer to reach a urea concentration of <2 M and then digested with 1 : 50 enzyme/substrate ratio of sequencing-grade modified trypsin (Promega). Then, 50 % trifluoroacetic acid (TFA) was added to lower the pH to 2 in order to stop the tryptic digest and to precipitate the *Rapi*GEST. Water-immiscible degradation products of *Rapi*GEST were pelleted by centrifugation at 12 000 ***g*** for 10 min. The cleared peptide solution was desalted with C18 reversed-phase columns (SepWPak Vac C18; Waters). The columns were pre-conditioned 2–3 times with acetonitrile and equilibrated three times with Buffer A (2 % acetonitrile, 0.1 % TFA in H_2_O) prior to sample loading. The flow-through was re-loaded onto the column and the column was then washed three times with Buffer A. The peptides were eluted from the column using Buffer B (50 % acetonitrile, 0.1 % TFA in H_2_O) and the elution step was repeated. The eluate was dried under vacuum using a rotary evaporator at 45 °C. Dried peptide pellets were re-suspended in MS buffer (2 % acetonitrile, 0.1 % TFA in ultrapure H_2_O) to a concentration of 1 µg µl^−1^, sonicated in a water bath for 3 min and supernatant was harvested by centrifugation at 12 000 ***g*** for 10 min.

SWATH MS measurements were conducted at the Institute for Molecular Systems Biology at ETH Zurich. One microgram of each peptide sample was measured in SWATH mode on a TripleTOF 5600 mass spectrometer using data-independent acquisition settings as described earlier [29, 38–40]. Resulting raw SWATH data were analysed using an automated pipeline and the software OpenSWATH with the *M. tuberculosis* H37Rv SWATH assay library [38]. Differential expression analysis of proteins identified in *M. tuberculosis* H37Rv and *M. bovis* AF2122/97 samples was dependent on the detection of the protein in both species. The differences in protein fold changes and the corresponding FDR corrections between *M. tuberculosis* H37Rv and *M. bovis* AF2122/97 were calculated using MSstats [39, 41]. A |log_2_FC|>0.56 and an FDR<0.05 was required for a protein to be defined as differentially expressed between *M. tuberculosis* H37Rv and *M. bovis* AF2122/97.

### Animals

All animal procedures were performed in accordance with the Irish Cruelty to Animals Act 1876 as amended by EU Directive 86/609/EEC with ethical approval from the University College Dublin (UCD) Animal Research Ethics Committee (AREC-13–14-Gordon). Ten unrelated Holstein-Friesian male calves (7–12 weeks old) were maintained under uniform housing conditions and nutritional regimens at the UCD Lyons Research Farm (Newcastle, County Kildare, Ireland). All animals were selected from a TB-free herd that is screened annually using the single intradermal comparative tuberculin skin test.

### Alveolar macrophage isolation, cell culture and infection

The laboratory methods used to isolate, culture and infect bovine alveolar macrophages with *M. bovis* AF2122/97 and *M. tuberculosis* H37Rv, and generate strand-specific RNA-seq libraries using RNA harvested from these cells have been described in detail by us elsewhere [15, 42]. An abridged description of the laboratory methods used in this study is provided below and the complete bioinformatics pipeline is accessible online (https://github.com/kerrimalone/AlvMac). Total lung cells were harvested by pulmonary lavage of lungs obtained post-mortem and stored in freezing solution [10 % DMSO (Sigma-Aldrich), 90 % FBS] at a density of 2.5×10^7^ cells ml^−1^ in 1 ml cell aliquots at −140 °C. When required, the cell pellet was resuspended in 15 ml of R10^+^ medium and placed in a 75 cm^2^ vented culture flask (CELLSTAR; Greiner Bio-One) and incubated for 24 h at 37 °C, 5 % CO_2_. After incubation, medium was removed together with non-adherent cells, adherent cells were washed with 15 ml Hanks balanced saline solution (HBSS) pre-warmed to 37 °C and dissociated by adding 10 ml pre-warmed 1× non-enzymatic cell dissociation solution (Sigma-Aldrich) to each culture flask. Cells were then pelleted (200 ***g*** for 5 min), resuspended in 10 ml pre-warmed R10^+^ medium and counted using a Vi-CELL XR Cell Viability Analyzer and reagent kit (Beckman Coulter). Mean viable cell recovery was estimated at ~80 % for each animal. Cell counts for each animal were adjusted to 5×10^5^ cells ml^−1^ (based on viable cell counts) using pre-warmed R10^+^ medium, seeded at 5×10^5^ cells per well on individual 24-flat-well tissue culture plates (Sarstedt) and incubated for a further 24 h at 37 °C, 5 % CO_2_, until required for mycobacterial infection. The purity of the seeded macrophages for each animal sample was 95 % as estimated by flow cytometry analysis (data not shown).

*M. bovis* AF2122/97 and *M. tuberculosis* H37Rv were cultured in Middlebrook 7H9-ADC medium containing either 0.2 % (v/v) glycerol for *M. tuberculosis* or 10 mM sodium pyruvate for *M. bovis* at 37 °C until mid-logarithmic phase (OD_600_ of 0.6–0.8). Prior to infection, mycobacterial cultures were pelleted by centrifugation (200 ***g***, 10 min), and pellets were disrupted with 3 mm sterile glass beads (Sigma-Aldrich) by vortexing at top speed for 1 min. Cells were resuspended in pre-warmed R10 medium, sonicated at full power (Branson Ultrasonics) for 1 min and the cell number was then adjusted to 5×10^6^ c.f.u. ml^−1^ (OD_600_ of 0.1=1×10^7^ c.f.u.) for an m.o.i. of 10 bacilli per alveolar macrophage.

For the infection time course, the R10 medium was removed from the macrophages and replaced with 1 ml R10 medium containing *M. bovis* AF2122/97 or *M. tuberculosis* H37Rv (5×10^6^ c.f.u. ml^−1^); parallel non-infected control alveolar macrophages received 1 ml R10 medium only. The alveolar macrophages were incubated at 37 °C, 5 % CO_2_,for periods of 2, 6, 24 and 48 h post-infection (p.i.). Following completion of the 2 h p.i. time point, the 2 h p.i. macrophages were lysed (by adding 250 µl RLT-1% β-mercaptoethanol buffer per tissue culture plate well) and stored at −80 °C, while the medium for the 6, 24 and 48 h p.i. macrophages was replaced with 1 ml fresh R10 medium per well and cells were reincubated at 37 °C, 5 % CO_2_, until required for harvesting. Colony-forming units were monitored over the infection time course (Fig. S1).

### RNA extraction, RNA-seq library preparation and high-throughput sequencing for bovine alveolar macrophage samples

For the current study, 127 strand-specific RNA-seq libraries were prepared. These comprised *M. bovis* AF2122/97-, *M. tuberculosis* H37Rv- and non-infected samples from each time point (0, 2, 6, 24 and 48 h) across 10 animals (with the exception of one animal that did not yield sufficient alveolar macrophages for *in vitro* infection at the 48 h p.i. time point). RNA extractions from macrophage lysates included an on-column genomic DNA elimination step (RNeasy Plus Mini kit; Qiagen). RNA quantity and quality was assessed using a NanoDrop 1000 spectrophotometer (Thermo Fisher Scientific) and a Bioanalyzer and an RNA 6000 Nano LabChip kit (Agilent Technologies). All samples displayed a 260 nm/280 nm absorbance ratio >2.0 and RIN values >8.5. In total, 200 ng total RNA from each sample was used for RNA-seq library preparation. Poly(A) mRNA enrichment was performed (Dynabeads mRNA DIRECT Purification Kit; Invitrogen, Life Technologies) and poly(A)-enriched mRNA was used to prepare individually barcoded strand-specific RNA-seq libraries (ScriptSeq version 2 RNA-Seq Library Preparation Kit; Illumina). The libraries were pooled into three sequencing pools and sequenced across 24 flow cell lanes (Illumina HiSeq2000; Beijing Genomics Institute).

### Differential gene expression analysis of bovine alveolar macrophage RNA-seq data

Computational analyses was performed on a 32-node Compute Server with Linux Ubuntu (version 12.04.2). Briefly, pooled libraries were deconvoluted, and adapter sequence contamination and paired-end reads of poor quality were removed. At each step, read quality was assessed with FastQC (version 0.10.1) [30]. Paired-end reads were aligned to the *Bos taurus* reference genome (*B. taurus* UMD3.1.1) with STAR aligner [43]. Read counts for each gene were calculated using featureCounts, set to unambiguously assign uniquely aligned paired-end reads in a stranded manner to gene exon annotation [32]. Differential gene expression analysis was performed using the edgeR Bioconductor package that was customized to filter out all bovine rRNA genes, genes displaying expression levels below 1 count per million (CPM) in at least ten individual libraries and identify DE genes between all pairs of infection groups within each time point, correcting for multiple testing using the Benjamini–Hochberg method with log_2_FC>1 and <−1 and an FDR threshold of significance <0.05 [44, 45]. Cellular functions and pathways over-represented in DE gene lists were assessed using the SIGORA R package [46].

## Results

### Differential RNA and protein expression between *M. bovis* AF2122/97 and *M. tuberculosis* H37Rv

For this study, 12 strand-specific RNA-seq libraries were prepared from *M. bovis* AF2122/97 (*n*=6) and *M. tuberculosis* H37Rv (*n*=6) grown exponentially in Sauton’s basal medium, pH 7.0 (Fig. S2; mapping statistics can be found in Table S2). An ‘Identical’/'Variable’ gene dataset was constructed in which orthologous genes between the two species were separated into those genes whose protein products are of equal length and 100 % conserved at the amino acid level (Identical, *n*=2775) from those that are not (Variable, *n*=1224) (Fig. 1a and Table S1). In total, 170 and 146 DE Identical genes and 133 and 124 DE Variable genes were identified for *M. bovis* AF2122/97 and *M. tuberculosis* H37Rv, respectively, amounting to 573 DE genes in total (Fig. 1a, b and Table S3). Twelve SWATH MS datasets were generated from total protein samples harvested from the same cultures as the RNA (Fig. S3 and Table S3). Overall, 2627 proteins were detected using the *M. tuberculosis* assay library (~70 % and~56 % of the total Identical and Variable proteins, respectively) (Fig. S3 and Table S3) [38, 40]. Of the 1937 Identical proteins detected by SWATH MS, 232 and 218 were found to be upregulated *M. bovis* AF2122/97 and *M. tuberculosis* H37Rv, respectively, totalling 450 DE proteins (Fig. 1a, b and Table S3). In total, 133 and 215 Variable proteins were found to be upregulated *M. bovis* AF2122/97 and *M. tuberculosis* H37Rv, respectively, amounting to 348 DE Variable proteins in total (50.4 %) (Fig. 1a, b and Table S3). Overlap of the DE lists for *M. bovis* AF2122/97 and *M. tuberculosis* H37Rv revealed 77 and 103 genes that are significantly upregulated in either species at both the RNA and the protein level, respectively (Fig. 1d); the top 20 of these are represented in Fig. 1(c).

Genes encoding MPB70 and MPB83 were the top two genes upregulated at the RNA and protein level in *M. bovis* AF2122/97; this is a result of a non-functional anti-SigK protein in *M. bovis* leading to constitutive upregulation of the SigK regulon, of which the *mpb83* and *mpb70* genes are components [47]. Furthermore, Rv0216/Mb0222, a double hotdog hydratase, is upregulated in *M. bovis* AF2122/97 at both the RNA and the protein level as previously observed by microarray analysis [27]. Amongst the genes upregulated at both the RNA and the protein level in *M. tuberculosis* H37Rv are: *ppe51*; antitoxin *vapB47*; and nitrate reductase-associated genes *narH* and *narG*, previously reported as upregulated at the RNA level in *M. tuberculosis* in comparison to *M. bovis* as a result of a SNP in the promoter region of *narGHJI* (Fig. 1c) [25, 26]. Incomplete overlap between DE genes at the transcriptional and translational level seen in this study has been reported in other studies and can be attributed to post-transcriptional and post-translational regulation within the cell, but also to more technical aspects, such as differences in detection limits and particular thresholds chosen to define DE RNA or protein [48–50].

### Transcription factor enrichment analysis: the PhoP regulon and ESX-1 secretion system

The differential gene expression observed between *M. bovis* AF2122/97 and *M. tuberculosis* H37Rv may be a consequence of differences in global transcriptional network regulation between the two species. To address this hypothesis, a formal transcription factor enrichment analysis was performed and revealed the significant association of 16 transcription factors with the DE Identical genes between *M. bovis* AF2122/97 (*n*=146) and *M. tuberculosis* H37Rv (*n*=170) (Fig. 1e) [37]. The association of transcription factors, such as alternate sigma factors SigK and SigF along with the cytoplasmic redox sensor WhiB3, with the DE gene lists indicates that disparate expression of virulence-related pathways regulated by these transcription factors between the two pathogens could have significant consequences for infection [47, 51, 52]. Furthermore, PhoP, EspR and DosR are also significantly associated with the DE genes; these transcription factors are important for adaptation of *M. tuberculosis* H37Rv to the intracellular environment and are functionally linked by such processes (Fig. 1e) [53, 54].

**Fig. 2.**
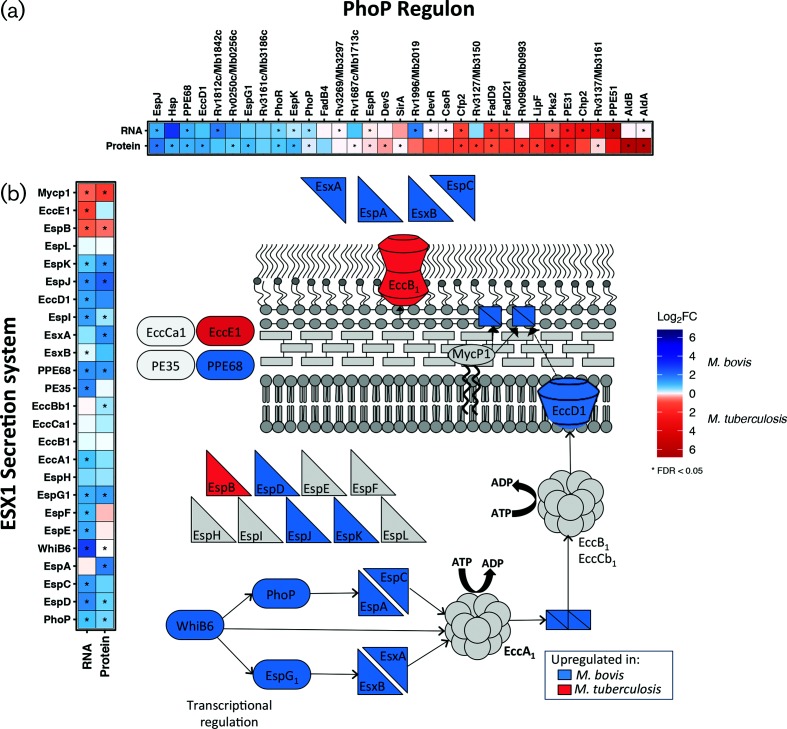
The DE genes [|log_2_FC|>1, FDR<0.05 (‘*’)] belonging to (a) the PhoP regulon and (b) the ESX-1 secretion system that are upregulated in *M. bovis* AF2122/97 (blue) or *M. tuberculosis* H37Rv (red). Inset: a representation of the ESX-1 secretion system pathway of *M. tuberculosis* coloured according to the upregulation of the associated gene in *M. bovis* AF2122/97 (blue) or *M. tuberculosis* H37Rv (red).

The PhoPR two-component system has a major role in regulating the pathogenic phenotype of *M. tuberculosis* by controlling the expression of a variety of virulence-associated pathways including SL-1, DAT and PAT lipid production and the Type-VII secretion system ESX-1; mutations in the PhoPR system of *M. bovis* have been suggested to play a role in the host specificity between the bovine- and human-adapted mycobacterial species [23, 53, 55–57]. Further investigation into the 72-gene regulon of PhoP identified 33 DE genes between the two species and these are presented in Fig. 2(a). The production of lipids SL-1 and PDIM is under PhoP regulation and is coupled within the *M. tuberculosis* cell; intriguingly *M. bovis* is reported to lack SL-1 in the cell envelope [58–60]. In this study, the expression of genes associated with the biosynthesis of SL-1 (e.g. *papA1*, *papA2*, *pks2*, *mmpL8*) was at a higher level in *M. tuberculosis* H37Rv and, conversely, genes associated with the biosynthesis of PDIM (e.g. *ppsA-E*, *lppX*) were expressed at a higher level in *M. bovis* AF2122/97 at the RNA and protein level (Fig. S4). SL-1 is one of the most abundant lipids in the mycobacterial outer membrane, is unique to pathogenic mycobacteria, is immunogenic and is implicated in the alteration of phago-lysosome fusion. Likewise, PDIM is required for mycobacterial virulence, facilitates macrophage invasion and protects against reactive nitrogen species. The differential expression of lipid-associated systems between the two species therefore presents distinct lipid-repertoires to interact with the host that could affect the overall success of infection [61–67].

The major antigens ESAT-6 and CFP10 are secreted by the ESX-1 system of *M. tuberculosis*, a system which has been implicated in mycobacterial escape from the phagosome to the cytosol that results in a Type I IFN response within the infected macrophage [57, 68, 69]. PhoP and EspR regulate the expression of ESX-1 secretion system-related genes and as stated are significantly associated with the DE genes between the two pathogens; despite EspR being expressed to a higher level in *M. tuberculosis* H37Rv (Table S3), there was a significant upregulation of the ESX-1 secretion system in *M. bovis* AF2122/97 in comparison to *M. tuberculosis* H37Rv, including ESX-1-related proteins such as EsxA, EspA, EspC and EspD at both the transcriptional and the translational level [55–57, 70, 71] (Fig. 2b). Mutation of the *whiB6* promoter in *M. tuberculosis* H37Rv leads to reduced PhoP regulation of ESX-1 secretion in this strain [56], while PhoP was expressed to a higher level in *M. bovis* AF2122/97; this latter observation may represent a compensatory mechanism for aberrant PhoP signalling and supports previous reports of suboptimal PhoP signalling in *M. bovis* [23, 59]. Seven of 55 genes regulated by DosR were expressed higher at the RNA level in *M. tuberculosis* H37Rv, probably reflecting the intimate relationship of DosR and its associated regulon with that of PhoP/EspR/WhiB3 (Table S4).

### A ‘core’ macrophage response common to infection with either species

In total, 127 strand-specific RNA-seq libraries were prepared from bovine alveolar infected macrophages that comprised *M. bovis* AF2122/97-, *M. tuberculosis* H37Rv- and non-infected samples from each time point (0, 2, 6, 24 and 48 h p.i.) across 10 animals, with the exception of one animal that did not yield sufficient alveolar macrophages for *in vitro* infection at the 48 h p.i. time point). Matched non-infected macrophage control samples were included for all infection time points (Fig. S2). Quality control and mapping statistics can be found in Table S5 and Fig. S5.

**Fig. 3.**
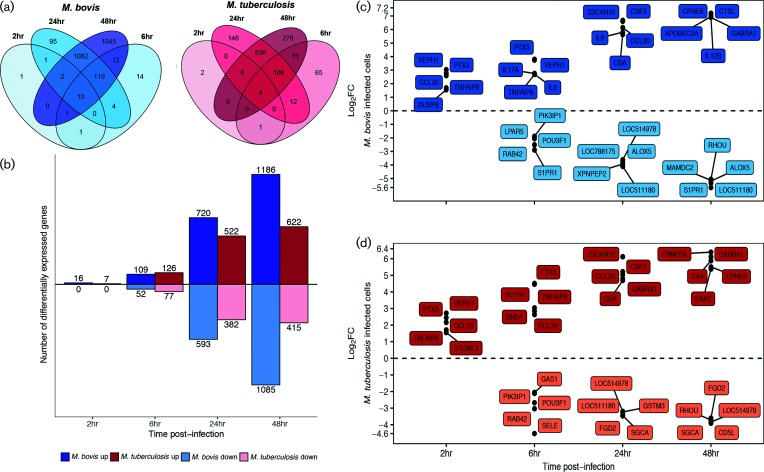
(a) The DE genes (|log_2_FC|>1, FDR<0.05) of bovine alveolar macrophages infected with *M. bovis* AF2122/97 (blue) or *M. tuberculosis* H37Rv (red) at 2, 6, 24 and 48 h p.i. (b) The number (*y*-axis) and direction of change (up=positive *y*-space, down=negative *y*-space) of DE genes (|log_2_FC|>1, FDR<0.05) of bovine alveolar macrophages infected with *M. bovis* AF2122/97 (blue) or *M. tuberculosis* H37Rv (red) at 2, 6, 24 and 48 h p.i. (*x*-axis). (c) The top five upregulated (positive *y*-space) and five downregulated (negative *y*-space) DE genes (|log_2_FC|>1, FDR<0.05) of bovine alveolar macrophages infected with *M. bovis* AF2122/97 or (d) *M. tuberculosis* H37Rv at 2, 6, 24 and 48 h p.i. (*x*-axis).

**Fig. 4.**
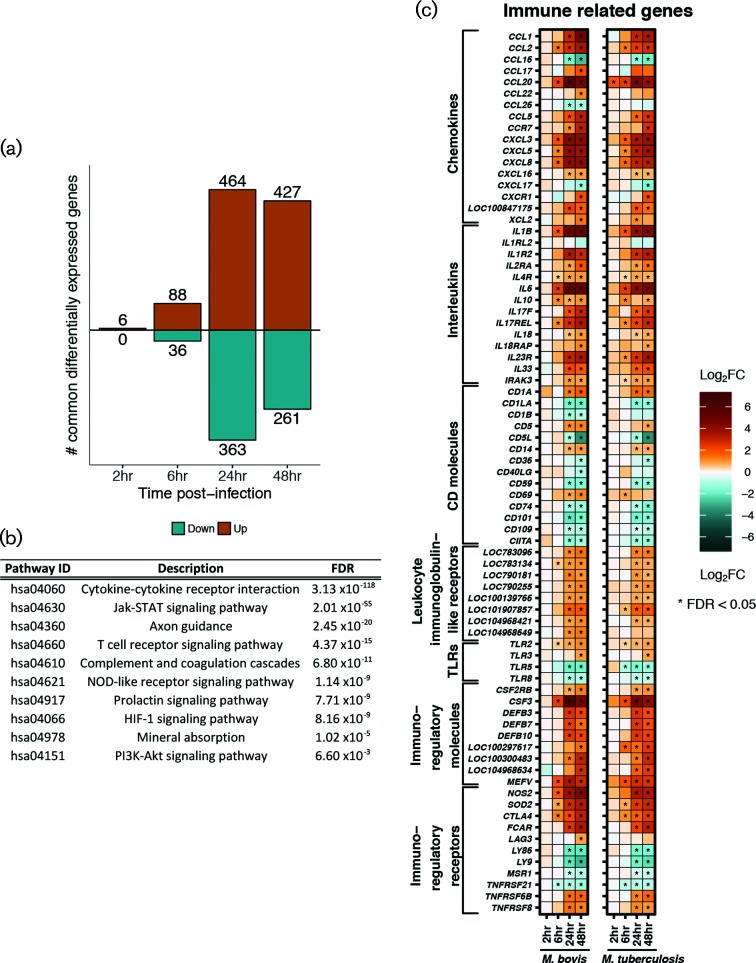
(a) The number (*y*-axis) and direction of change (up=orange, down=cyan) of genes that are commonly differentially expressed (‘core response’) (|log_2_FC|>1, FDR<0.05, with non-significant delta comparison values) in bovine alveolar macrophages infected with *M. bovis* AF2122/97 and infected with *M. tuberculosis* H37Rv at 2, 6, 24 and 48 h p.i. (b) Pathways enriched for 688 genes that are commonly differentially expressed (‘core response’) in bovine alveolar macrophages infected with *M. bovis* AF2122/97 and *M. tuberculosis* H37Rv over the first 24 h of infection (FDR<0.05). (c) Genes that are commonly differentially expressed (‘core response’) [|log_2_FC|>1, FDR<0.05 (‘*’)] and associated with the innate immune response in bovine alveolar macrophages infected with *M. bovis* AF2122/97 (left column) or *M. tuberculosis* H37Rv (right column) over 48 h p.i.

The comparison of *M. bovis* AF2122/97*-* or *M. tuberculosis* H37Rv*-*infected macrophages with respect to non-infected macrophages revealed a sequential increase in the number of DE genes across the infection time course, which peaked at 48 h p.i. and a larger number of DE genes were seen in *M. bovis* AF2122/97*-*infected macrophages with the exception of at 6 h p.i. (Fig. 3a–c and Table S6); similar temporal expression profiles were previously reported in other *in vitro* bovine and human macrophage infection studies [42, 72–74]. Comparison of these DE gene lists identified a subset of genes that displayed the same directionality and a similar magnitude of expression (Fig. 4a and Table S7). The association of enriched pathways such as *Cytokine-cytokine receptor interaction*, *NOD-like receptor signalling* and *Jak-STAT signalling* with this gene subset suggests a robust ‘core’ macrophage response to infection with either mycobacterial species throughout the time course (Fig. 4b). The core response includes numerous key genes known to be involved in the innate immune response against pathogenic mycobacteria such as: *CCL20* [75]; *IL18*, which limits the growth of *M. tuberculosis* in human macrophages [76–78]; anti-inflammatory *IL10* [79]; and *NOS2*, polymorphisms of which are associated with susceptibility of Holstein cattle to bovine TB [80] (Fig. 4c). Furthermore, the *HIF-1* signalling pathway was significantly enriched for the DE genes common to both infection series; this pathway is associated with regulating a switch in central glucose metabolism during high-energy demanding events, such as infection, in neutrophils and macrophages [81] (Fig. S6a).

### DNA sensing and RIG-I like signalling pathways are found in the divergent response to infection with *M. tuberculosis* H37Rv and *M. bovis* AF2122/97

**Fig. 5.**
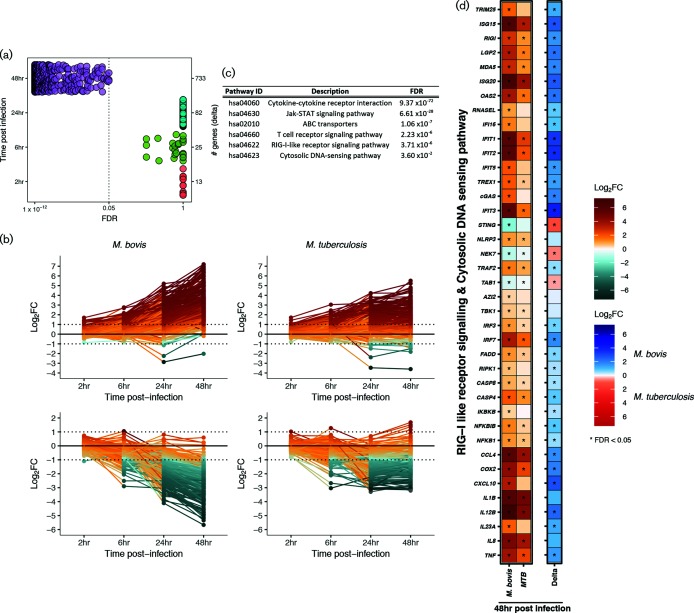
(a) The number of genes (right *y*-axis) that are changing (|log_2_FC|>1) and that pass the FDR threshold (FDR<0.05) from the comparative analysis of *M. bovis* AF2122/97*-* or *M. tuberculosis* H37Rv*-*infected macrophages in contrast to control macrophages and subsequently in contrast to the other infection series (delta comparison) at 2, 6, 24 and 48 h p.i. [‘# genes (delta)’]. (b) Line graphs representing those DE functionally annotated genes (*n*=576) that exhibit a higher magnitude of change in *M. bovis* AF2122/97*-*infected macrophages versus *M. tuberculosis* H37Rv*-*infected macrophages in a positive manner (*n*=323) (left and right top panel, respectively) and in a negative manner (*n*=253) (left and right bottom panel, respectively) at 2, 4, 24 and 48 h p.i. (c) Pathways that are enriched for 576 functionally annotated genes that exhibit divergent expression patterns in *M. bovis* AF2122/97*-* or *M. tuberculosis* H37Rv*-*infected macrophages at 48 h p.i. (FDR<0.05). (d) The DE genes (|log_2_FC|>1, FDR<0.05) associated with *RIG-I*-*like* and *DNA sensing signalling* pathways in bovine alveolar macrophages infected with *M. bovis* AF2122/97 (blue) or *M. tuberculosis* H37Rv (red) at 48 h p.i.

Aside from the defined ‘core’ response genes, there were larger numbers of DE genes in *M. bovis* AF2122/97-infected macrophages in contrast to *M. tuberculosis* H37Rv infection, mainly at 24 h p.i. (1313 versus 904 genes, respectively) and 48 h p.i. (2271 versus 1037 genes, respectively) (Fig. 3a, b). Comparison of the relative change with respect to control between *M. bovis* AF2122/97*-* and *M. tuberculosis* H37Rv*-*infected macrophages at each time point revealed a statistically significant divergence in their responses at 48 h p.i. only associated with DE signatures from 703 genes (Fig. 4a and Table S8). Analysis of the expression pattern of 576 of these genes with functional annotation across time revealed a greater magnitude of change in *M. bovis* AF2122/97*-*infected macrophages, where DE genes are up- or down-regulated to a higher extent in *M. bovis* AF2122/97*-*infected macrophages or not significantly changed at all in *M. tuberculosis-*infected macrophages (Fig. 5c). Pathway enrichment analysis revealed association of *ABC transporters* with the divergent annotated gene set (Fig. 5b); these are involved in cholesterol efflux from the cell, and manipulation of host cell cholesterol transport and metabolism has been documented in *M. tuberculosis-*containing macrophages [82]. A general dampening in the expression of cholesterol-associated genes was noted in *M. bovis* AF2122/97*-*infected macrophages at 48 h p.i. (Fig. S6b). Pathway enrichment analysis also associated *Cytosolic DNA-sensing* and *RIG-I-like receptor signalling* with the 576 divergent genes (Fig. 5c). Type I IFNs have been associated with pathogenesis during *M. tuberculosis* infection and their production has been found to be dependent on the mycobacterial ESX-1 secretion system and the cytosolic sensing of extracellular *M. tuberculosis* DNA and subsequent cGAS-STING-dependent signalling [83–86].

**Fig. 6.**
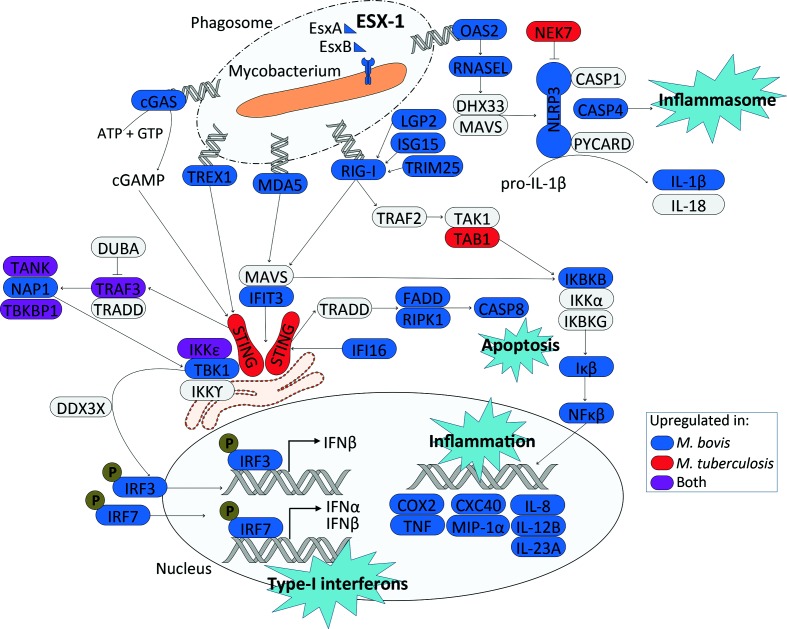
An overview of the DNA sensing and RIG-I signalling identified in this study 48 h after infection of bovine alveolar macrophages with *M. bovis* AF2122/97 and *M. tuberculosis* H37Rv. Blue and red represent upregulation of the associated gene in either *M. bovis* AF2122/97*-* or *M. tuberculosis* H37Rv-infected macrophages, respectively, while purple represents upregulation of the associated gene in both infection models. The ESX-1 secretion system was upregulated in *M. bovis* AF2122/97 in comparison to *M. tuberculosis* H37Rv.

Overall, there is a stronger upregulation of genes encoding proteins involved in *RIG-I-like* and *DNA-sensing signalling* in *M. bovis-*infected macrophages in comparison with *M. tuberculosis* H37Rv*-*infected macrophages at 48 h p.i. These include genes encoding DNA sensors such as MB21D1/cGAS, MDA5, IFI16 and DDX58/RIG-I, antiviral and MAVS-TBK1 interacting protein IFIT3, serine/threonine kinase TBK1, and key IFN transcriptional regulators IRF3 and IRF7 that are all known to contribute to STING-dependent induction of type I IFNs (Figs 5d and 6) [87–89]. Furthermore, genes *LGP2*, *ISG15* and *TRIM25* that encode regulators of *DDX58*/*RIGI* gene expression are upregulated in *M. bovis*-infected macrophages at 48 h p.i. [90]. Likewise, downstream of RIG-I, genes *IKBKB* and *IKB* are also expressed to a higher level in *M. bovis* AF2122/97-infected macrophages along with *NFKB*, the gene encoding a key transcription factor that regulates the expression of inflammatory-related genes [89]. Targets of NFKB such as *TNF*, *COX2*, *CXC40*, *MIP1a*, *IL8*, *IL12* and *IL23a* are all expressed to a higher degree in *M. bovis* AF2122/97-infected macrophages with respect to *M. tuberculosis* H37Rv-infected macrophages at 48 h p.i. (Figs 5d and 6). A low level of reads mapped to the type I IFN genes *IFNAD* (orthologue of human *IFNA1*) and *IFNB1*, and only in a subset of animals at certain time points, excluding these genes from DE analysis based on filtering criteria.

Independent of RIG-I signalling, genes involved in *DNA sensing* such as *TREX1*, which encodes a 3′−5′ exonuclease that senses and degrades cytosolic DNA to prevent type I IFN production through the TBK1/STING/IRF3 pathway, and *OAS2* were also expressed to a higher level in *M. bovis-*containing macrophages [91]. OAS2 is a dsRNA binding protein that generates 2′−5′-adenosine oligomers which activate RNase L resulting in the assembly of the NLRP3 inflammasome and IL-1b production [92, 93]; genes *RNASEL*, *NLRP3* and non-canonical activation of the NLRP3 inflammasome *CASP4* were all found to be expressed to a higher level in *M. bovis* AF2122/97-infected macrophages at 48 h p.i. [94, 95] (Figs 4c and 6). Taken together, these data highlight divergence in the engagement of *M. bovis* AF2122/97 and *M. tuberculosis* H37Rv with the nucleic acid sensing system of the bovine macrophage, which in turn would influence downstream immune-related events, and ultimately infection outcome.

## Discussion

The data we present here provide insight into the molecular basis of host tropism between *M. tuberculosis* and *M. bovis* in the bovine host. We have taken advantage of both RNA-seq and SWATH MS to compare the global transcriptional and translational expression profiles of *M. tuberculosis* H37Rv and *M. bovis* AF2122/97 for definition of functional variation between the two species that may more broadly explain the host preference exhibited between human and bovine tubercle bacilli. We focused on *M. tuberculosis* H37Rv and *M. bovis* AF2122/97 as they are widely used reference strains and they have been previously used to demonstrate the attenuation of *M. tuberculosis* in the bovine host [1, 9]. Other studies have assessed in isolation either the transcriptome by microarray or the proteome by shotgun MS [27, 28, 96, 97]; the resolution afforded by both RNA-seq and SWATH MS in comparison with previous studies has allowed for the most complete dataset for *M. bovis* to date and the most complete comparative dataset between *M. bovis* and *M. tuberculosis*.

The relatively small number of DE genes at both the RNA and the protein level between *M. bovis* AF2122/97 and *M. tuberculosis* H37Rv highlights the close genetic relationship between the two pathogens. That said, assessment of these DE genes supports our hypothesis that subtle genetic changes between the two species result in divergent phenotypes driven by differential expression of major virulence-associated factors and pathways. We found that transcription factors PhoP, WhiB3 and DosR are significantly associated with the DE genes between the two species and these are functionally linked by processes that govern the adaptation of *M. tuberculosis* to the intracellular environment [53, 54]. The PhoPR two-component system is important for *M. tuberculosis* infection and it has been suggested that mutations in PhoR attenuate animal-adapted *M. bovis* in humans [23, 98–104]. PhoP regulates the production of virulence-associated cell wall lipids and controls the expression of EspA, an ESX-1 secretion pathway-related protein involved in the secretion of the major antigen EsxA/ESAT6 [55, 56, 70, 71]. We found that the ESX-1 secretion system was expressed to a higher degree at both the RNA and the protein level in *M. bovis* AF2122/97 in comparison with *M. tuberculosis* H37Rv; differences in ESX-1 secretion system expression between the two pathogens may be a consequence of a SNP in the promoter of the *whiB6* gene in *M. tuberculosis* H37Rv or attributed to attenuated PhoPR signalling in *M. bovis* [56]. There is an emerging body of evidence showing that *M. tuberculosis* can rupture the phagosome membrane through the action of the ESX-1 secretion system and that the activation of cytosolic DNA-sensing pathways and the production of Type I IFNs is dependent on ESX-1 expression [83–86]. Alternate transcriptional regulation between *M. tuberculosis* H37Rv and *M. bovis* AF2122/97 may represent differential priming events in preparation for the initial interactions of both species with their respective host immune systems. Increased expression of the ESX-1 secretion system may facilitate faster escape of *M. bovis* AF2122/97 from the phagosome into the cytosol in contrast to *M. tuberculosis* H37Rv, hence triggering DNA-sensing pathways and increased IFN response seen in our data [68, 85, 105].

To determine the impact of pathogen variation on host response, we conducted an experimental infection of primary bovine alveolar macrophages with *M. tuberculosis* H37Rv and *M. bovis* AF2122/97 and tracked the transcriptional response to infection. Although conducting a dual RNA-seq study would facilitate simultaneous assessment of the transcriptional response of both the host macrophage and invading mycobacteria during infection, this technique is limited with regard to the proportion of the bacterial-to-host transcriptome ratio in the resulting data [106]. The bovine alveolar macrophage response to infection with either pathogen was strikingly similar over the first 24 h of infection, reflected in similar intracellular c.f.u. numbers over the first 24 h (Fig. S1). Notably, a ‘core’ macrophage response displayed enrichment for DE genes involved in pathogen recognition, innate cell signalling, and cytokine and chemokine production, illustrating the initiation of host innate defence mechanisms in response to infection with *M. bovis* and *M. tuberculosis*. One of the most striking observations is that significant divergence in macrophage gene expression profiles between *M. bovis* AF2122/97 and *M. tuberculosis* H37Rv infections only occurred after 24 h. At 48 h p.i., enrichment for *DNA sensing* was found for 576 annotated genes that show divergent expression patterns between the two infection models. The innate immune system detects exogenous nucleic acid within the cell through pattern recognition receptors (PRRs) that include Absent in Melanoma 2 (AIM2)-like receptors (ALRs) with pyrin and HIN domains (PYHIN proteins), for example IFI16 [107–109]. Other DNA-sensing proteins include cytosolic RIG-I-like receptors (RLRs) (e.g. RIG-I, MDA5, LGP2), exonucleases, synthetases and cyclic GMP-AMP synthases (e.g. TREX1, OAS2 and cGAS) [83, 91, 93, 110, 111]. An increased transcriptional induction of genes associated with cGAS-STING-dependent signalling was seen in macrophages infected with *M. bovis* AF2122/97, including *MB21D1/cGAS* and downstream effectors *TBK1* and *IRF3* (Fig. 6). cGAS has been shown to have a central role during *M. tuberculosis* infection; 48–72 h p.i. cGAS senses cytosolic *M. tuberculosis* and in turn signals through STING to drive type I IFN production (Fig. 6) [68, 83–86, 112]. Surprisingly, the cGAS-STING axis was not the only PRR pathway found upregulated during mycobacterial infection, as the *RIG-I like signalling* pathway was also observed to be enriched at 48 h p.i., with genes encoding TREX1, OAS2, and RLRs RIG-I, MDA5 and LGP2 also found expressed to a higher level in *M. bovis* AF2122/97-infected macrophages. The identification of an increase in expression of DNA-sensing-related pathways in *M. bovis* AF212/97-infected macrophages at 48 h p.i. correlates with the differential expression of the ESX-1 secretion system between the two pathogens. A further role for the ESX-1 secretion system in host–pathogen interactions has been described through the activation of the NLRP3 inflammasome and the production of IL-1β [113–116]. Transcriptional signals associated with the NLRP3 inflammasome were higher in *M. bovis* AF2122/97-infected macrophages at 48 h p.i. along with the increased expression of *CASP4*, an NLRP3 inflammasome activator, which has a central role in mediating the response to *Legionella*, *Yersinia* and *Salmonella* bacterial infections in primary human macrophages and that has been found upregulated in the necrotic granuloma model of mice and lymph nodes of TB patients [92, 117–119]. Together, these data therefore suggest that not only does mycobacterial infection in the bovine macrophage trigger an increase in the transcription of the cGAS/STING/IRF3 pathway previously characterized as responsible for type I IFN production during *M. tuberculosis* infection, it also triggers alteration in the transcription of genes encoding auxiliary DNA sensing RLRs including RIG-I, MDA5 and TREX1, that likewise converge to signal through the STING complex (Fig. 6). Our data show that *M. bovis* AF2122/97 drove a stronger transcriptional response in the aforementioned pathways in the bovine alveolar macrophage at 48 h p.i. in comparison to *M. tuberculosis* H37Rv; studies with additional *M. bovis* strains will be needed to determine whether this represents a conserved relationship between *M. bovis* and the bovine host.

The upregulation of the ESX-1 secretion system at both the RNA and the protein level in *M. bovis* AF2122/97 with the observed upregulation of DNA-sensing pathways and the NLRP3/IL-1β pathway in *M. bovis* AF2122/97*-*infected macrophages shows how the expression level of virulence factors between MTBC species, rather than their presence or absence, can drive divergent host responses and influence infection outcome overall. This supports the hypothesis of divergent expression of virulence factors between *M. bovis* and *M. tuberculosis* playing a central role in host tropism. Indeed, the idea of a ‘balance’ with regard to the expression of mycobacterial virulence factors is reflected in the findings that production of IFN-β1 in monocyte-derived macrophages is strain-dependent amongst *M. tuberculosis* lineages [120]. That said, we cannot disregard that differences between the bovine and human host will play a factor. As the innate immune response in different mammals can vary, diversity in the expression and structure in key innate immune genes and engagement with pathogen factors must play major roles in host specificity and the outcome of pathogen encounter [121]. In this regard, it is interesting to note that the bovine PYHIN locus contains only *IFI16* (bovine PYHIN) and cattle are the only mammals to date found to encode a single member of the PYHIN protein family; in contrast, humans have four genes, and mice 13 genes [122]. Furthermore, polymorphisms in *NLRP3* have been found to influence host susceptibility to *M. tuberculosis* infection, its induction is associated with the mycobacterial ESX-1 secretion system, and bovine and human NLRP3 proteins share 83 % sequence similarity [123]. Further comparative studies of human and bovine immune genetics will aid in unravelling the complex differential host response to infection with both pathogens. Moreover, we cannot overlook the potential role of other secreted proteins, transport systems, cell wall lipids or factors that show quantitative differences in expression between *M. bovis* and *M. tuberculosis.* Indeed, MBP83 and MBP70 and additional components of the SigK regulon show constitutive upregulation in *M. bovis* versus *M. tuberculosis,* while pathways such as ESX-3 and Mce-1 were also found to be differentially expressed between the two pathogens at both the transcriptional and the translational level during *in vitro* growth in this study (Table S3) [47, 124]. The next step will be to extend our observations across multiple *M. bovis* and *M. tuberculosis* strains to define how quantitative species-specific differences translate to host tropism.

In conclusion, we established that *M. tuberculosis* H37Rv and *M. bovis* AF2122/97 induce divergent responses in infected bovine alveolar macrophages, a consequence of the differential expression of key mycobacterial virulence-associated pathways. Our work demonstrates the specificity of mycobacterial host–pathogen interaction and indicates how the subtle interplay between the phenotype of the invading mycobacteria and the subsequent host response may underpin host specificity amongst members of the MTBC.

## Supplementary Data

1396 Supplementary File 1

## Supplementary Data

496 Supplementary File 2

## Supplementary Data

16 Supplementary File 3

## Supplementary Data

1125 Supplementary File 4

## Supplementary Data

29 Supplementary File 5

## Supplementary Data

32 Supplementary File 6

## Supplementary Data

8880 Supplementary File 7

## Supplementary Data

801 Supplementary File 8

## Supplementary Data

94 Supplementary File 9
