# Supplementary material for: Comparative 'omics analyses differentiate Mycobacterium tuberculosis and Mycobacterium bovis and reveal distinct macrophage responses to infection with the human and bovine tubercle bacilli
Source: Microb Genom. 2018 Mar 20;4(3):e000163. doi: 10.1099/mgen.0.000163 (PMC5885015; doi:10.1099/mgen.0.000163)
Supplement: Supplementary File 1 [file mgen-4-163-s001.pdf]

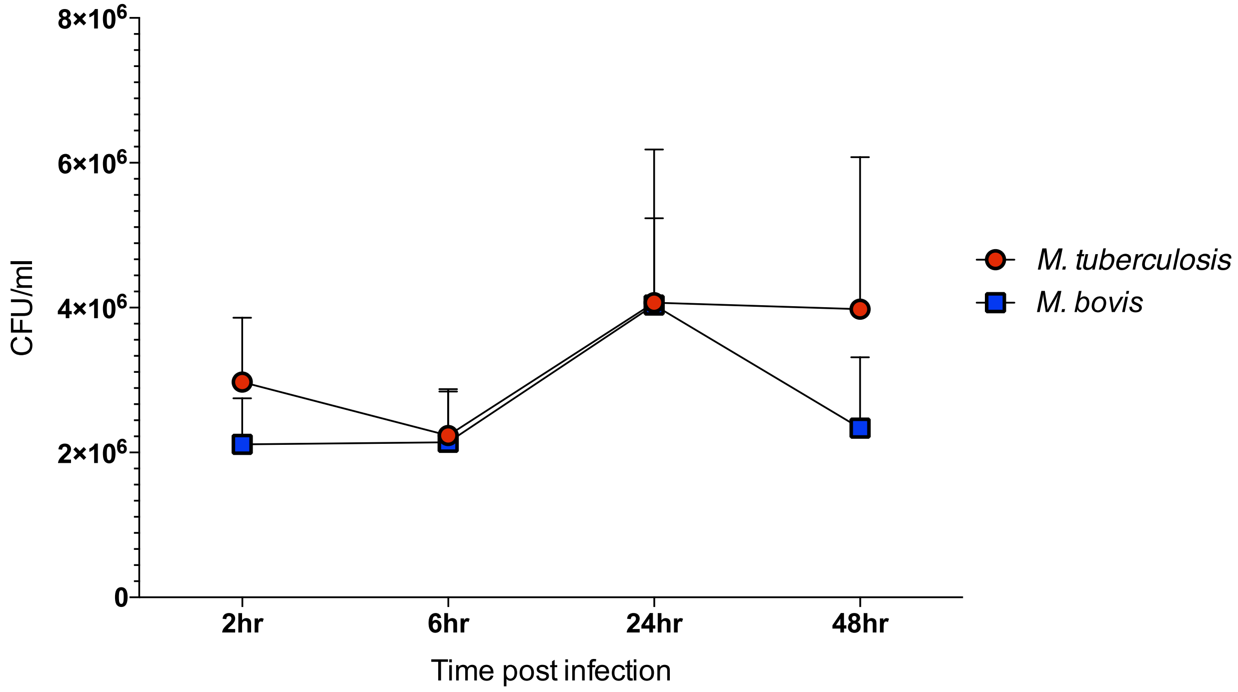

Figure S1: The number of colony forming units ('CFU/ml') recovered from bovine alveolar macrophages infected with *M. bovis* AF2122/97 (blue) or *M. tuberculosis* H37Rv (red) at 2, 6, 24 and 48 hours post-infection. (Error bars represent standard error of the mean, *n* = 6)

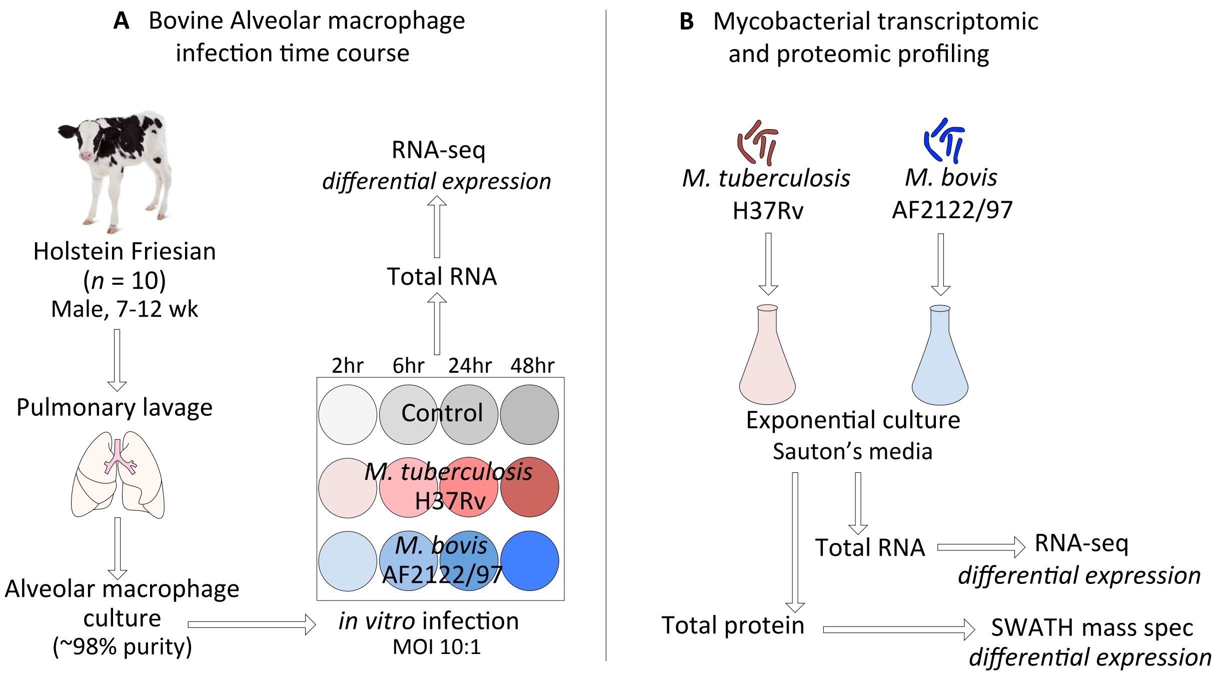

Figure S2: Overview of experimental design for the **A)** bovine alveolar macrophage infection time course and **B)** mycobacterial transcriptomic and proteomic profiling performed for this study.

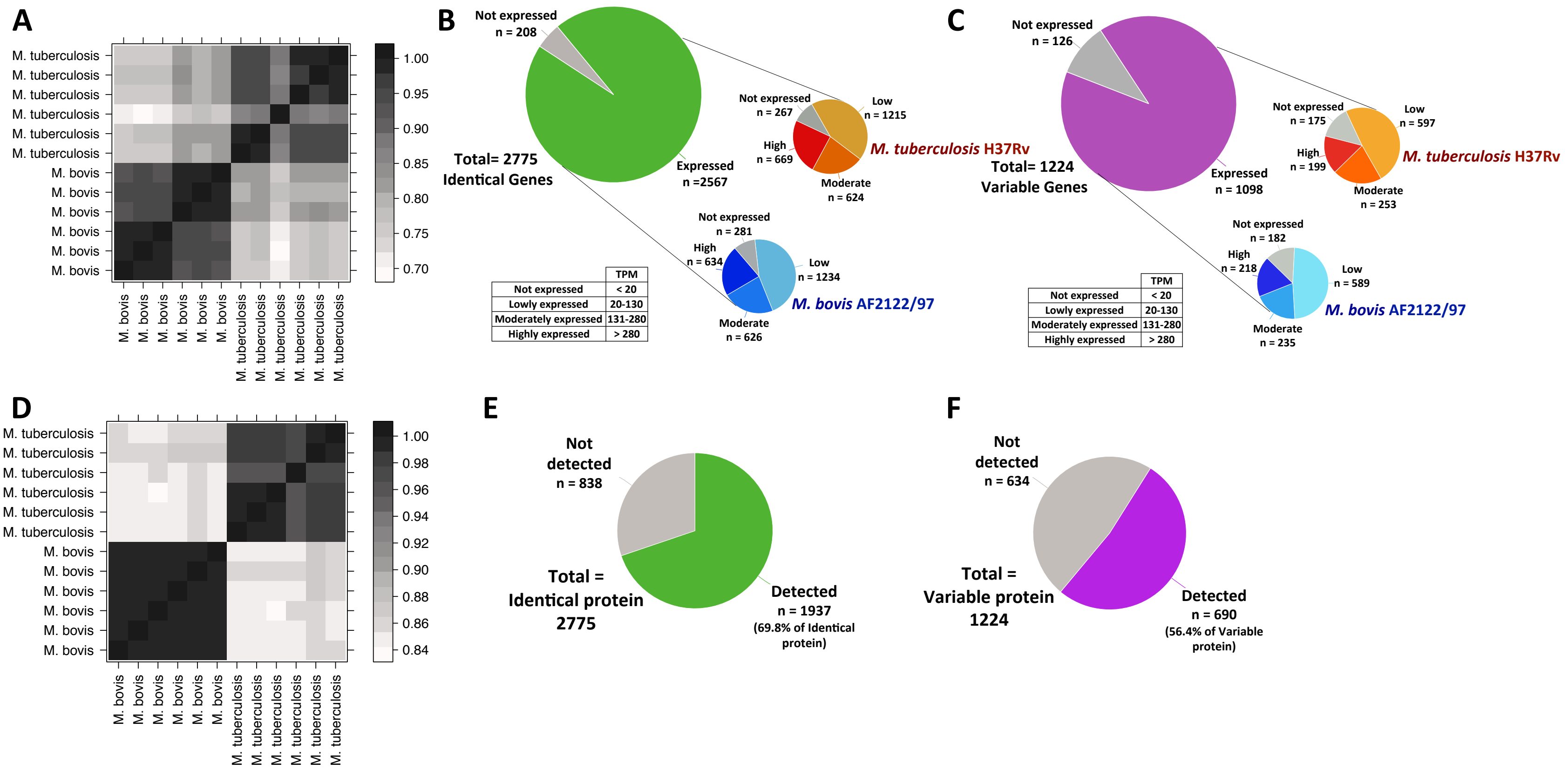

Figure S3: A) Pearson correlation plot of reads mapped to 2,775 Identical genes (100% conserved in length and amino acid sequence between the two species) in the six *M. bovis* AF2122/97 and six *M. tuberculosis* H37Rv RNA-seq datasets. Pie charts representing the proportion of **B)** Identical (100% conserved in length and amino acid sequence between the two species, green) and **C)** Variable genes (< 100% conserved in length and amino acid sequence between the two species, purple) detected and not detected across *M. bovis* AF2122/97 and *M. tuberculosis*

H37Rv RNA-seq datasets. RNA expression values (Transcripts per Million (TPM)) were calculated for each gene and gene expression within either species was categorised into not expressed (<20 TPM), lowly expressed (20-130 TPM), moderately expressed (131-280 TPM) and highly expressed (>280 TPM). **D)** Pearson correlation plot of the intensity values of the 2,627 identified proteins in the six *M. bovis* AF2122/97 and six *M. tuberculosis* H37Rv SWATH MS datasets. Pie charts representing the expression of **E)** 2775 Identical genes (100% conserved in length and amino acid sequence) and **F) 1**224 Variable genes (< 100% conserved in length and amino acid) in *M. bovis* AF2122/97 and *M. tuberculosis* H37Rv detected by SWATH MS.

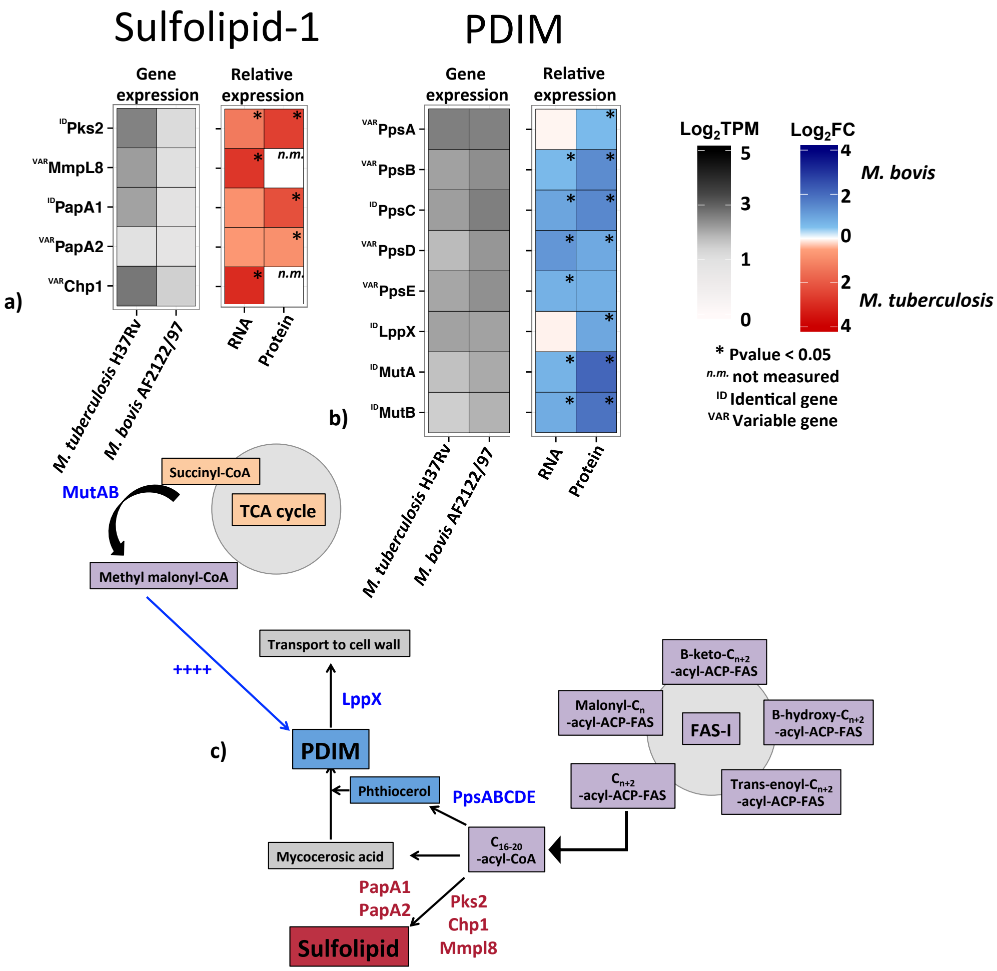

Figure S4: The expression of the **a)** sulfolipid-1 (SL-1) and **b)** phthiocerol dimycocerosate (PDIM) synthesis associated genes at the RNA and protein level in *M. tuberculosis* H37Rv (red) and *M. bovis* AF2122/97 (blue). The expression of each gene ("Gene expression") is presented as Log_{10}TPM at the RNA level while the relative expression ("Relative expression") between the two species is presented as log2FC. Those genes that change significantly at the RNA and protein level (FDR < 0.05) are denoted ('*'). **c)** Diagrammatic overview of the SL-1 and PDIM biosynthesis pathways in *M. tuberculosis*. Blue represents the genes in b) that are upregulated in *M. bovis* AF2122/97 in contrast to *M. tuberculosis* H37Rv and red represents the genes in a) that are upregulated in *M. tuberculosis* H37Rv.

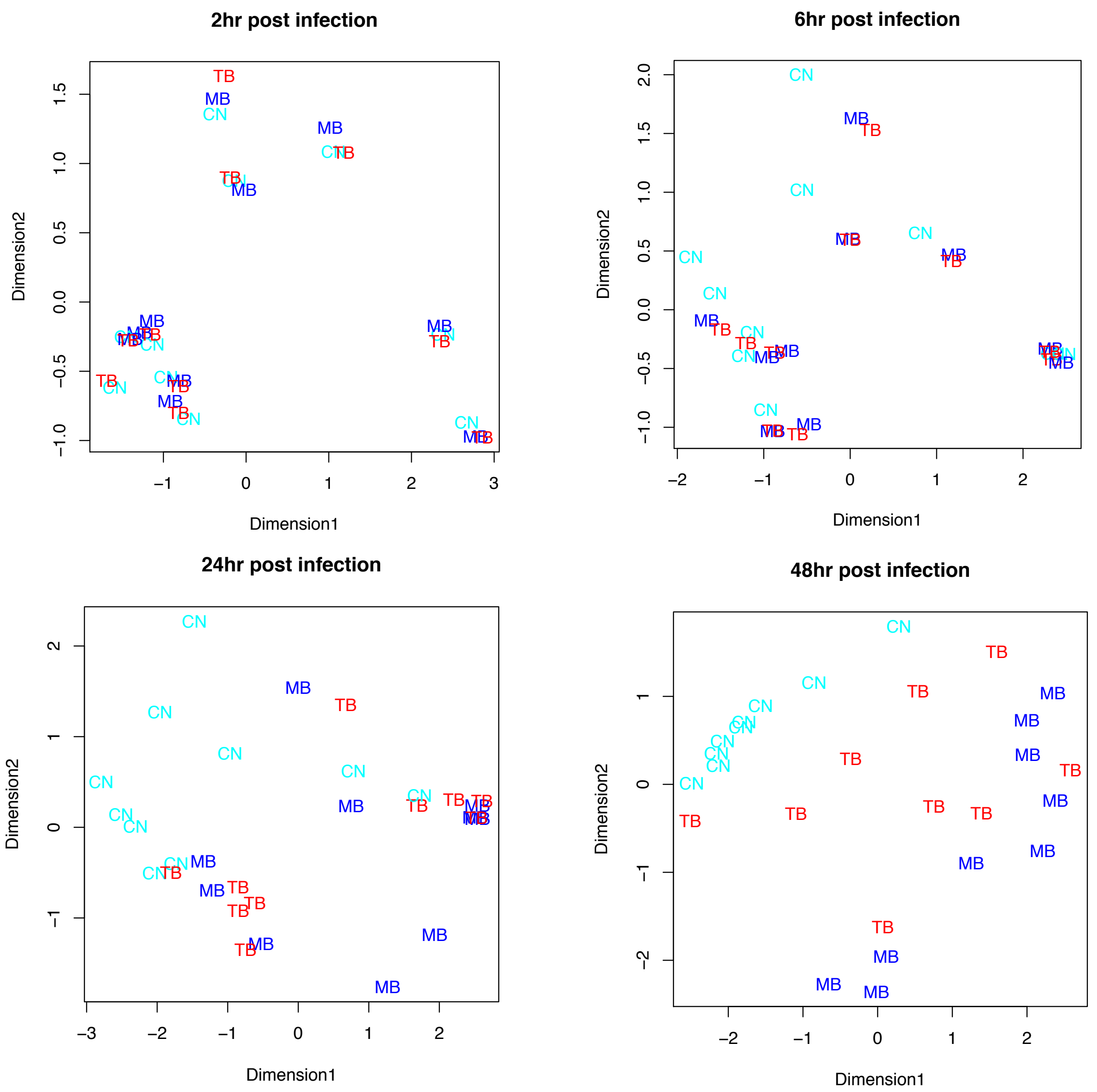

Figure S5: Multidimensional scaling plots of the RNA-seq expression data for individual of bovine alveolar macrophages infected with *M. bovis* AF2122/97 (‘MB’, blue), *M. tuberculosis* H37Rv (‘TB’, red) or none (‘CN’, cyan) at 2, 6, 24 and 48 hours post-infection.

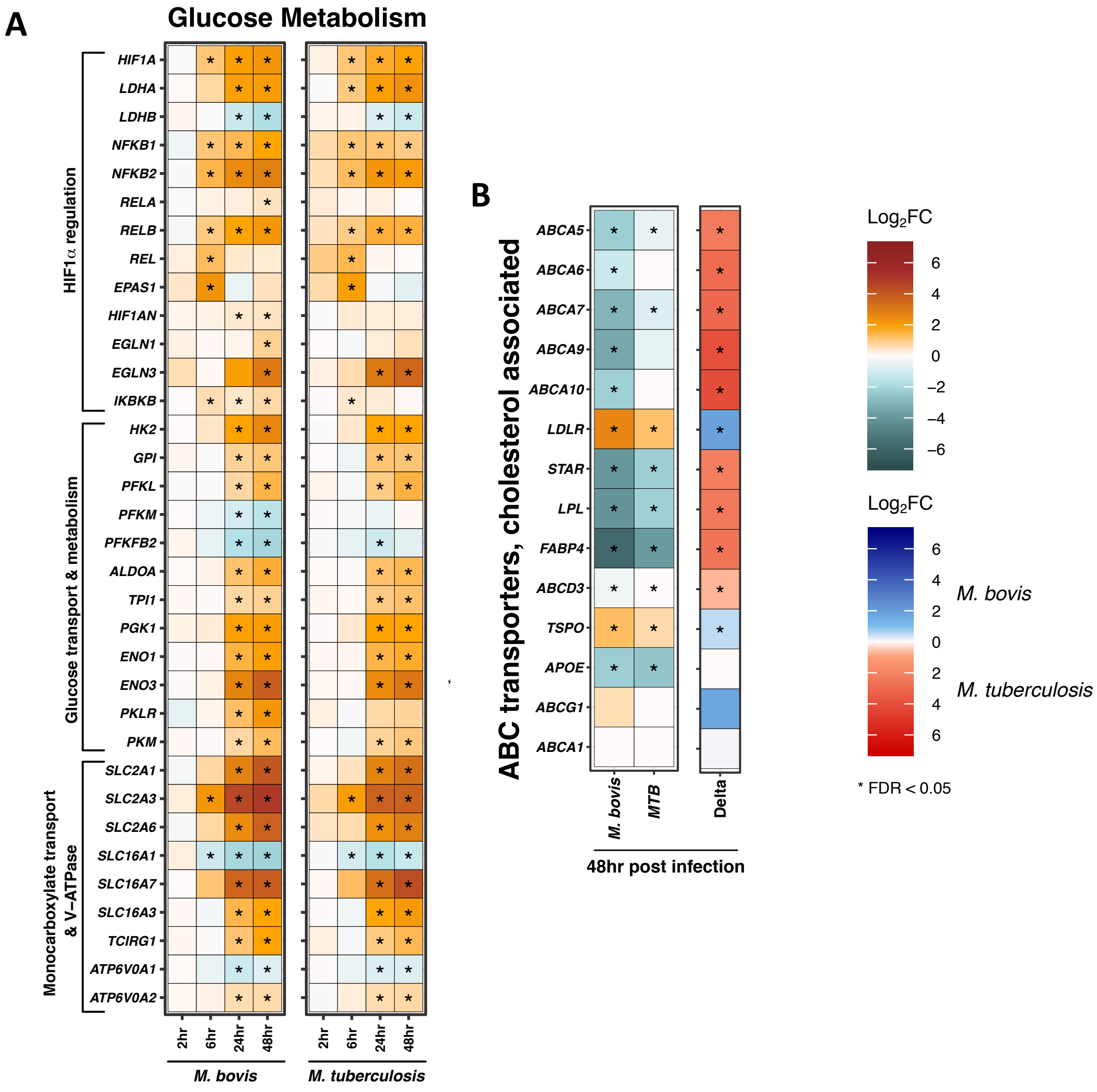

Figure S6: The expression of differentially expressed genes (|Log_2FC| > 1, FDR < 0.05 ('*') associated with **A)** glucose metabolism in bovine alveolar macrophages infected with *M. bovis* AF2122/97 or *M. tuberculosis* H37Rv ("MTB") at 2, 6, 24 and 48 hours post-infection and **B)** cholesterol-associated transport in bovine alveolar macrophages at 48 hours post-infection. Delta comparison shows genes upregulated in *M. bovis* AF2122/97 in blue and *M. tuberculosis* H37Rv in red.
